# Unraveling the Tapestry of Depression: A Cross-Sectional Study

**DOI:** 10.7759/cureus.66987

**Published:** 2024-08-16

**Authors:** Rohankumar Gandhi, Ilesh Kotecha, Kaushikkumar R Damor, Yogesh Murugan

**Affiliations:** 1 Community and Family Medicine, Shri Meghaji Pethraj (MP) Shah Government Medical College, Jamnagar, IND; 2 Community Medicine, Gujarat Medical Education and Research Society (GMERS) Medical College, Rajpipla, IND; 3 Family Medicine, Guru Gobind Singh Government Hospital, Jamnagar, IND

**Keywords:** sociodemographic factors, mental health, risk factors, urban slums, elderly, depression

## Abstract

Background: An often-occurring and severely disabling mental illness that mostly affects older people living in urban slums is depression. Developing successful therapies requires an understanding of the complex interactions between the different factors that contribute to depression in this susceptible population.

Objectives: This study aimed to find the prevalence of depression and identify the factors associated with depression in the geriatric population aged ≥60 years in the study area during the study period in Gujarat, India.

Methods: This study was carried out among 450 participants aged ≥60 years. Face-to-face interviews and standardized assessment tools, including the Geriatric Depression Scale (GDS) for depression and the Mini-Cog test for cognitive impairment, were used to collect data on depression levels, sociodemographic characteristics, behavioral factors, medical conditions, life events, and psychiatric history. Statistical analyses, including chi-squared tests, were performed to assess the associations.

Results: Significant associations were found between various factors and depression levels, which were lower education (11.11% severe depression among non-literate vs. 2.11% among literate, p<0.001) and widowhood (11.56% severe depression among widowed vs. 4.53% among married, p<0.001), which were linked to higher depression severity. Behavioral risk factors like short sleep duration (<6 hours at night: 21.71% severe depression, p<0.001), tobacco snuffing (16.24% severe depression, p<0.001), and lack of physical activity (28.71% severe depression, p<0.001) were strongly associated with increased depression. Medical conditions such as hypertension (10.36% severe depression, p<0.001) and stressful life events like family conflicts (16.67% severe depression, p<0.001) exhibited strong associations. A personal history of depression (38.82% severe depression, p<0.001) was a potent predictor.

Conclusions: The study highlights the multifaceted nature of depression in the geriatric population of the study area, underscoring the necessity of all-encompassing measures to tackle the recognized possible risk factors. The results provide valuable insights for developing targeted prevention strategies, healthcare policies, and support systems to enhance the mental well-being of this vulnerable population.

## Introduction

Depression affects millions of people globally, especially the elderly, and is a common and crippling mental health issue. A person's aging process can be exacerbated or precipitated by a variety of obstacles, such as deteriorating physical health, losing autonomy, a lack of social interaction, and life-changing events. Given its links to greater rates of illness and death, worse quality of life, and increased functional impairment, the burden of depression among the elderly is especially worrisome [1,2].

Globally, the depression prevalence among the elderly population varies widely, with estimates ranging from 4.7% to 16% in community-dwelling older adults [1,2]. However, because of the cumulative impact of socioeconomic status, suffering, inadequate access to healthcare services, an increased level of stress and adversity, and living in slum communities or financially disadvantaged areas, some subgroups of the elderly population may be more susceptible to depression.

Elderly depression has been linked to a number of causes. Age, gender, marital status, degree of education, and socioeconomic status are sociodemographic characteristics that have been repeatedly linked to the occurrence and severity of depression [3,4]. Furthermore, a higher incidence of depression in older persons has been associated with behavioral risk factors, such as inactivity, substance abuse (alcohol and tobacco), and sleep disorders [5-7].

Chronic medical illnesses, including diabetes, musculoskeletal disorders, cardiovascular diseases, and hypertension, have been demonstrated to have a reciprocal association with depression, whereby each condition may exacerbate the other [8,9]. In addition, important life events that occur in the elderly population, like the death of a loved one, financial hardships, or substantial life changes, can trigger depressive episodes [3,10].

Significantly, it has been found that a history of depression or other mental conditions in oneself or one's family can strongly predict the presence of depressive symptoms in an older adult [10,11]. This emphasizes how crucial it is to evaluate and treat depression in the geriatric population while taking into account the person's past mental health history as well as any possible hereditary or environmental predispositions.

Despite the expanding corpus of studies on depression in the senior citizen population, there remains a need for comprehensive studies that examine the interplay of various sociodemographic, behavioral, medical, and psychosocial factors in diverse settings, particularly among vulnerable populations residing in urban slums or low-resource areas. In order to improve the mental health and general quality of life of the senior population, specific measures and healthcare strategies can be informed by an understanding of the individual risk factors and factors that contribute to the onset of depression.

The current study sought to determine whether different risk factors for behavioral problems, medical diseases, life events, historical history of depression or psychiatric issues, and other sociodemographic variables were associated with higher levels of depression in older people living in urban slums. In order to provide important insights into the intricate interactions between determinants that lead to depression in this susceptible population, this study examined all of these variables in a community-based setting. The findings will ultimately help develop early identification, mitigation, and efficient management strategies for depressive disorders in the elderly.

## Materials and methods

Study design and setting

The research was planned to be a cross-sectional survey done in urban slums within the study area, using a community-based approach in Gujarat, India. The study duration spanned from November 2021 to December 2022.

Eligibility criteria

Inclusion Criteria

The geriatric population aged ≥60 years, residing in the study area for the past year, and providing consent to participate in the study were included.

Exclusion Criteria

Those individuals who did not give consent, those with a Mini-Cog test score <3 (suggestive of cognitive impairment or dementia), and those with pre-diagnosed psychiatric illnesses other than depression (such as anxiety, paranoid disorders, delusions, hallucinations, schizophrenia, etc.) were excluded. These exclusions were made to ensure reliable data collection and assessment of depression.

Sampling technique

At a 95% confidence level and a 5% absolute allowed error, the sample size was determined using an anticipated prevalence rate of 31% for geriatric depression. An estimated 342 individuals would be the required minimum sample size; however, to consider a 20% non-response rate, the sample size was expanded to 410 subjects [12].

The sampling technique involved a simple random sampling method, with the house as the primary sampling unit. All 27 urban slums in the study area were included, and from each slum, 16 elderly individuals were selected. The slums were divided into four quadrants, and in each quadrant, houses were chosen at random using a random number table. Using the Mini-Cog test, all eligible elderly people in the chosen homes were evaluated for cognitive impairment and, if they satisfied the eligibility requirements, were enrolled in the study.

Sampling method

In-person interviews with the qualified senior participants were done at their residences as part of the data-gathering procedure. Prior to starting the interviews, topic specialists' opinions were sought in order to determine the questionnaire's content validity. Additionally, a pilot study was conducted in an area other than the main study area to standardize the questionnaire and identify potential operational difficulties.

The questionnaire was rendered into the regional language, Gujarati, and then back into English to guarantee that the questions' meanings stayed the same. The participants' literacy status was questioned during the interviews, and literate individuals were defined as those who could read and write with understanding in any language.

Data collection tool

The data collection tool was a pre-tested, semi-structured questionnaire designed to gather information from the elderly participants. The questionnaire consisted of the following components: (1) sociodemographic factors such as age, sex, religion, education, occupation, marital status, income, socioeconomic status (modified Brahm Govind (BG) Prasad classification) [13], house ownership (own/rented), family size, number of children, type of family, financial dependency, and health insurance status; (2) assessment scales, namely, (a) Mini-Cog test to assess cognitive impairment [14] and (b) GDS-30 Scale (Geriatric Depression Scale using 30 items) to assess depression levels (score: 0-9: no depression; 10-19: mild depression; and 20-30: severe depression) [15]; (3) behavioral factors such as sleep duration, alcohol consumption, tobacco use (chewing and snuffing), smoking habits, and physical activity levels; (4) medical conditions such as information on chronic diseases or conditions the participant was suffering from; (5) life events such as significant life events experienced by the participant in the past year; and (6) history of depression such as information on the participants and their family members' history of depression or other psychiatric problems.

Data analysis

Microsoft Excel was used for data entry, and IBM SPSS Statistics for Windows, V. 22.0 (IBM Corp., Armonk, NY) was used for statistical analysis. To analyze the data, appropriate statistical tests were applied. For finding the association between various sociodemographic variables, behavioral risk factors, medical conditions, life events, and depression levels, the chi-squared test was used. The significance of the associations was determined based on the calculated p-values. The data was presented and summarized using frequencies and percentages. A significance level of p-value <0.05 was applied.

Ethical consideration

Ethical considerations were duly addressed, such as obtaining approval from the Institutional Ethics Committee of Shri Meghaji Pethraj (MP) Shah Government Medical College and Guru Gobind Singh Government Hospital, Jamnagar (approval number: 123/05/2021), ensuring voluntary participation, maintaining confidentiality, and obtaining informed consent.

## Results

Various sociodemographic risk factors and depression were described in Table 1. It shows the association between various sociodemographic risk factors and depression among the study participants. Age was found to have a statistically significant association (p=0.04) with depression; those aged 75-84 years (15.25%) and ≥85 years (11.11%) were more likely to experience severe depression than those aged 60-74 years (5.77%). Depression and education level were also significantly associated (p<0.001), with a higher incidence of severe depression among illiterate people (11.11%) than among literate people (2.11%). Marital status showed a highly significant association with depression (p<0.001), with the highest proportion of severe depression observed among unmarried, separated, or divorced individuals (18.75%), followed by widows and widowers (11.56%) and married individuals (4.53%). The study found a significant association between depression and the monthly income of the family (p<0.001) as well as socioeconomic level (p=0.024). Notably, individuals with lower incomes and socioeconomic positions were more likely to experience severe depression. A higher percentage of people who live in rented homes (18.92%) than in homes they own (6.29%) experience severe depression. The ownership status of a home was also substantially correlated with depression (p=0.0067). Depression had a significant association with the number of children (p=0.0267), with a greater rate of severe depression (12.33%) among those without children than among those with 1-2 children (6.49%) or more than two children (5.80%). Furthermore, a higher number of those without health insurance (10.21%) experienced severe depression compared to those with health insurance (4.18%), and having health insurance was substantially associated with lower levels of depression (p=0.03).

**Table 1:** Association between sociodemographic characteristics and depression p<0.05: significant; p<0.001: highly significant; BG: Brahm Govind

Variables	Category		Depression			Total	Chi-squared (ꭓ^2^)value	P-value (p)
			No depression	Mild depression	Severe depression			
Age (in years)	60-74		245 (67.31%)	98 (26.92%)	21 (5.77%)	364 (80.89%)	ꭓ^2^=12.387	p=0.04
	75-84		30 (50.85%)	20 (33.90%)	9 (15.25%)	59 (13.11%)		
	≥85		13 (48.15%)	11 (40.74%)	3 (11.11%)	27 (6.00%)		
Gender	Male		126 (63.96%)	61 (30.96%)	10 (5.08%)	197 (43.78%)	ꭓ^2^=3.08	p=0.21
	Female		162 (64.03%)	68 (26.88%)	23 (9.09%)	253 (56.22%)		
Religion	Hindu		212 (65.84%)	85 (26.40%)	25 (7.76%)	322 (71.55%)	ꭓ^2^=3.657 (with Yates' correction)	p=0.45
	Muslim		74 (61.16%)	40 (33.06%)	7 (5.78%)	121 (26.89%)		
	Christian		2 (28.57%)	4 (57.14%)	1 (14.28%)	7 (1.56%)		
Education (literacy)	Literate		137 (72.49%)	48 (25.40%)	4 (2.11%)	189 (42.00%)	ꭓ^2^=16.976	p<0.001
	Not literate		151 (57.86%)	81 (31.03%)	29 (11.11%)	261 (58.00%)		
Occupation (employment)	Employed		109 (69.40%)	42 (26.12%)	7 (4.48%)	158 (35.11%)	ꭓ^2^=4.113	p=0.13
	Unemployed		179 (61.30%)	87 (29.79%)	26 (8.91%)	292 (64.89%)		
Marital status	Married		213 (74.22%)	61 (21.25%)	13 (4.53%)	287 (63.78%)	ꭓ^2^=39.878 (with Yates' correction)	p<0.001
	Widow/widower		73 (49.66%)	57 (38.78%)	17 (11.56%)	147 (32.67%)		
	Unmarried/separated/divorced		2 (12.50%)	11 (68.75%)	3 (18.75%)	16 (3.55%)		
Family monthly income (₹=in rupees)	≤₹ 10,000		167 (58.19%)	91 (31.71%)	29 (10.10%)	287 (63.78%)	ꭓ^2^=15.035	p<0.001
	>₹ 10,000		121 (74.23%)	68 (23.31%)	4 (2.46%)	163 (36.22%)		
Socioeconomic status (modified BG Prasad's classification 2022)	I (upper)		14 (87.50%)	2 (12.50%)	0 (0.00%)	16 (3.55%)	ꭓ^2^=17.64 (with Yates' correction)	p=0.024
	II (upper middle)		31 (77.50%)	6 (15.00%)	3 (7.50%)	40 (8.89%)		
	III (middle)		78 (72.90%)	24 (22.43%)	5 (4.67%)	107 (23.78%)		
	IV (lower middle)		90 (60.40%)	51 (34.23%)	8 (5.37%)	149 (33.11%)		
	V (lower)		75 (54.35%)	46 (33.33%)	17 (12.32%)	138 (30.67%)		
House ownership status	Own		271 (65.62%)	116 (28.09%)	26 (6.29%)	413 (91.78%)	ꭓ^2^=10.021	p=0.007
	Rented		17 (45.95%)	13 (35.13%)	7 (18.92%)	37 (8.22%)		
Family size	1-5		161 (65.72%)	64 (26.12%)	20 (8.16%)	245 (54.45%)	ꭓ^2^=3.417	p=0.69
	6-10		105 (62.50%)	51 (30.36%)	12 (7.14%)	168 (37.33%)		
	>10		22 (59.46%)	14 (37.84%)	1 (2.70%)	37 (8.22%)		
No. of children	0		39 (53.42%)	25 (34.25%)	9 (12.33%)	73 (16.22%)	ꭓ^2^=10.987	p=0.03
	1-2		211 (68.51%)	77 (25.00%)	20 (6.49%)	308 (68.45%)		
	>2		38 (55.07%)	27 (39.13%)	4 (5.80%)	69 (15.33%)		
Type of family	Nuclear		102 (62.96%)	47 (29.01%)	13 (8.03%)	162 (36.00%)	ꭓ^2^=2.497	p=0.65
	Joint		89 (63.57%)	44 (31.43%)	7 (5.00%)	140 (31.11%)		
	Three generation		97 (65.54%)	38 (25.68%)	13 (8.78%)	148 (32.89%)		
Extent of financial dependency	Independent		49 (68.05%)	20 (27.78%)	3 (4.17%)	72 (16.00%)	ꭓ^2^=2.753	p=0.59
	Partially dependent		71 (65.14%)	32 (29.36%)	6 (5.50%)	109 (24.22%)		
	Totally dependent		168 (62.45%)	77 (28.63%)	24 (8.92%)	269 (59.78%)		
Type (on whom) of financial dependency		Self	49 (68.05%)	20 (27.78%)	3 (4.17%)	72 (16.00%)	ꭓ^2^=7.241 (with Yates' correction)	p=0.29
		Spouse	21 (70.00%)	6 (20.00%)	3 (10.00%)	30 (6.67%)		
		Children	209 (64.51%)	92 (28.40%)	23 (7.09%)	324 (72.00%)		
		Distant family members	9 (37.50%)	11 (45.83%)	4 (16.67%)	24 (5.33%)		
Health insurance		With health insurance	146 (67.91%)	60 (27.91%)	9 (4.18%)	215 (47.78%)	ꭓ^2^=6.626	p=0.03
		No health insurance	142 (60.43%)	69 (29.36%)	24 (10.21%)	235 (52.22%)		

Table 2 shows the various behavioral risk factors and depression. The associations between several behavioral risk variables and study participants' levels of depression are analyzed in this table. Sleep duration, both during the day and at night, showed a highly significant association with depression (p<0.001 for both). A higher proportion of individuals with sleep durations of less than one hour during the day (13.82%) and less than six hours at night (21.71%) experienced severe depression compared to those with longer sleep durations. Also, there was a significant association (p<0.001) between tobacco addiction and depression. Among those who snuffed tobacco, the prevalence of severe depression was highest (16.24%), followed by chewers (7.19%) and non-users (1.20%). On the other hand, there was a stronger correlation between smoking addiction and depression (p<0.001) among smokers, with 11.84% reporting severe depression compared to 6.42% among non-smokers. Physical activity/exercise for at least 30 minutes per day showed a highly significant association with depression (p<0.001). Serious depression was more common in those who did not exercise (28.71%) than in those who worked out three times a week (3.75%) or every day (0.37%). Depression and alcohol addiction were also significantly associated (p<0.001) with a larger percentage of alcohol addicts (11.25%) reporting severe depression than non-addicts (6.49%).

**Table 2:** Various behavioral risk factors and depression p<0.05: significant; p<0.001: highly significant

Behavioral risk factors	Category	Depression			Total	Chi-squared (ꭓ^2^) value	P-value (p)
		No depression	Mild depression	Severe depression			
Sleep duration (in hours) in a daytime	<1 hr	71 (32.72%)	116 (53.46%)	30 (13.82%)	217 (48.22%)	ꭓ^2^=171.762 (with Yates' correction)	p<0.001
	1-3 hrs	168 (92.82%)	10 (5.52%)	3 (1.66%)	181 (40.22%)		
	>3 hrs	49 (94.23%)	3 (5.77%)	0 (0.00%)	52 (11.56%)		
Sleep duration (in hours) in a nighttime	<6 hrs	4 (3.10%)	97 (75.19%)	28 (21.71%)	129 (28.67%)	ꭓ^2^=299.156 (with Yates' correction)	p<0.001
	6-8 hrs	25 (62.50%)	12 (30.00%)	3 (7.50%)	40 (8.89%)		
	>8 hrs	259 (92.17%)	20 (7.12%)	2 (0.71%)	281 (62.44%)		
Tobacco addiction	Chewing	92 (55.09%)	63 (37.72%)	12 (7.19%)	167 (37.11%)	ꭓ^2^=99.551	p<0.001
	Snuffing	44 (37.61%)	54 (46.15%)	19 (16.24%)	117 (26.00%)		
	No	152 (91.57%)	12 (7.23%)	2 (1.20%)	166 (36.89%)		
Smoking addiction	Yes	28 (36.84%)	39 (51.32%)	9 (11.84%)	76 (16.89%)	ꭓ^2^=29.437	p<0.001
	No	260 (69.52%)	90 (24.06%)	24 (6.42%)	374 (83.11%)		
Physical activity/exercise for at least 30 minutes/day	Daily	252 (93.68%)	16 (5.95%)	1 (0.37%)	269 (59.78%)	ꭓ^2^=296.458 (with Yates' correction)	p<0.001
	Thrice weekly	32 (40.00%)	45 (56.25%)	3 (3.75%)	80 (17.78%)		
	No	4 (3.96%)	68 (67.33%)	29 (28.71%)	101 (22.44%)		
Alcohol addiction	Yes	18 (22.50%)	53 (66.25%)	9 (11.25%)	80 (17.78%)	ꭓ^2^=76.16	p<0.001
	No	270 (72.97%)	76 (20.54%)	24 (6.49%)	370 (82.22%)		

Table 3 shows the known cases of various medical conditions and depression. This table examines the association between known cases of various medical conditions and depression levels among the study participants. Depression was significantly associated with hypertension (p<0.001), asthma (p<0.001), arthritis/musculoskeletal issues (p<0.001), vision impairment (p<0.001), any cardiac illness (p<0.001), chronic constipation (p<0.001), and other unidentified conditions (p<0.001). For these conditions, a higher proportion of individuals with the condition experienced mild or severe depression compared to those without the condition. As an illustration, among individuals with hypertension, the percentages of mild and severe depression were 39.84% and 10.36%, respectively, whereas among those without hypertension, they were lower. Furthermore, the absence of any comorbidities was found to be substantially linked with lower levels of depression (p<0.001), as all individuals in this group reported not experiencing any depression.

**Table 3:** Known cases of various medical conditions and depression p<0.05: significant; p<0.001: highly significant

Known cases of medical conditions (multiple responses)	Depression			Total	Chi-squared (ꭓ^2^)value	P-value (p)
	No depression	Mild depression	Severe depression			
Diabetes	42 (54.55%)	28 (36.36%)	7 (9.09%)	77/450 (17.11%)	ꭓ^2^=3.609	p=0.16
Hypertension	125 (49.80%)	100 (39.84%)	26 (10.36%)	251/450 (55.78%)	ꭓ^2^=49.685	p<0.001
Asthma	5 (21.74%)	15 (65.22%)	3 (13.04%)	23/450 (5.11%)	ꭓ^2^=19.138 (with Yates' correction)	p<0.001
Arthritis/musculoskeletal problems	166 (52.70%)	119 (37.78%)	30 (9.52%)	315/450 (70.00%)	ꭓ^2^=58.231	p<0.001
Visually impaired	8 (25.00%)	19 (59.38%)	5 (15.62%)	32/450 (7.11%)	ꭓ^2^=19.88	p<0.001
Hearing impaired	15 (60.00%)	6 (24.00%)	4 (16.00%)	25/450 (5.56%)	ꭓ^2^=2.979	p=0.23
Any cardiac disease	5 (13.16%)	22 (57.89%)	11 (28.95%)	38/450 (8.44%)	ꭓ^2^=50.506	p<0.001
Chronic constipation	4 (25.00%)	10 (62.50%)	2 (12.50%)	16/450 (3.56%)	ꭓ^2^=8.888 (with Yates' correction)	p<0.001
Communicable disease (TB/HIV, etc.)	9 (50.00%)	9 (50.00%)	0 (0.00%)	18/450 (4.00%)	ꭓ^2^=3.152 (with Yates' correction)	p=0.21
Any other	13 (21.31%)	35 (57.38%)	13 (21.31%)	61/450 (13.56%)	ꭓ^2^=59.184	p<0.001
No comorbidities	80 (100.00%)	0 (0.00%)	0 (0.00%)	80/450 (17.78%)	ꭓ^2^=50.506 (with Yates' correction)	p<0.001
Comorbidities	208 (56.22%)	129 (34.86%)	33 (8.92%)	370/450 (82.22%)		

Table 4 shows the various life events in the past one year and depression. This table shows the relationship between the research participants' depression levels and several life events that occurred within the last year. Each of the listed life events, family conflicts (p<0.001), unemployment of self or children (p<0.001), illness (p<0.001), illness of family members (p<0.001), family member or close relative death (p<0.001), big purchases or home construction (p<0.001), and financial problems or losses (p<0.001), was significantly linked to depression. In comparison to those who did not experience the event, there is a greater percentage of people who did report mild or severe depression for each of these life events. For instance, 57.78% of people with mild depression and 16.67% of people with severe depression were among those who encountered family conflicts; the equivalent percentages were lower among those who did not. Overall, having any of the life events on the list during the previous year was substantially linked to greater levels of depression (p<0.001), whereas having none of these events was linked to lower levels of depression.

**Table 4:** Various life events in the past year and depression p<0.05: significant; p<0.001: highly significant

Various life events in the past one year (multiple responses)	Depression			Total	Chi-squared (ꭓ^2^)value	P-value (p)
	No depression	Mild depression	Severe depression			
Conflicts in family	23 (25.55%)	52 (57.78%)	15 (16.67%)	90/450 (20.00%)	ꭓ^2^=72.601	p<0.001
Unemployment of self or children	6 (20.00%)	16 (53.33%)	8 (26.67%)	30/450 (6.67%)	ꭓ^2^=32.929	p<0.001
Illness of self	52 (30.59%)	88 (51.76%)	30 (17.65%)	170/450 (37.78%)	ꭓ^2^=133.786 (with Yates' correction)	p<0.001
Illness of family members	69 (53.08%)	43 (33.08%)	18 (13.84%)	130/450 (28.89%)	ꭓ^2^=15.223	p<0.001
Death of family members	15 (31.92%)	24 (51.06%)	8 (17.02%)	47/450 (10.44%)	ꭓ^2^=24.342	p<0.001
Death of close relatives	8 (36.36%)	10 (45.46%)	4 (18.18%)	22/450 (4.89%)	ꭓ^2^=8.747 (with Yates' correction)	p=0.013
Big purchase or construction of home	68 (43.31%)	67 (42.68%)	22 (14.01%)	157/450 (34.89%)	ꭓ^2^=47.301	p<0.001
Financial problems or loss	185 (55.56%)	116 (34.83%)	32 (9.61%)	333/450 (74.00%)	ꭓ^2^=40.318 (with Yates' correction)	p<0.001
Any of the above	241 (60.40%)	126 (31.58%)	32 (8.02%)	399/450 (97.11%)	ꭓ^2^=17.69 (with Yates' correction)	p<0.001
None of the above	47 (92.16%)	3 (5.88%)	1 (1.96%)	51/450 (2.89%)		

Table 5 shows past history of depression/psychiatric problems and depression. This table examines the relationship between research participants' or their families' past history with depression or other mental issues and depression. A past history of depression (p<0.001), receiving treatment for depression in the past (p<0.001), any other psychiatric problem in the past (p<0.001), and having parents, siblings, or children who suffered from depression in the past (p<0.001) were all highly significantly associated with current depression levels. Individuals who had a positive history of any of these characteristics were more likely than those who had not to report having mild to severe depression. For instance, among individuals who had previously experienced depression, 60% had mild depression and 38.82% had severe depression; in contrast, the comparable percentages were significantly lower among those who had never had depression. In general, higher levels of current depression were substantially (p<0.001) associated with any of the listed factors related to a past history of depression or psychiatric issues, whereas lower levels of depression were connected with none of these factors.

**Table 5:** Past history of depression/psychiatric problems and depression p<0.05: significant; p<0.001: highly significant

History of depression/psychiatric problems (multiple responses)	Depression			Total	Chi-squared (ꭓ^2^)value	P-value (p)
	No depression	Mild depression	Severe depression			
Did you suffer from depression in the past?	1 (1.18%)	51 (60.00%)	33 (38.82%)	85/450 (18.89%)	ꭓ^2^=234.437 (with Yates' correction)	p<0.001
Did you take treatment for depression in the past?	0 (0.00%)	6 (50.00%)	6 (50.00%)	12/450 (2.67%)	ꭓ^2^=33.083 (with Yates' correction)	p<0.001
Any other psychiatric problems in the past?	0 (0.00%)	8 (38.10%)	13 (61.90%)	21/450 (4.67%)	ꭓ^2^=95.269 (with Yates' correction)	p<0.001
Did your parents, siblings, or children suffer from depression in the past?	0 (0.00%)	10 (55.56%)	8 (44.44%)	18/450 (4.00%)	ꭓ^2^=44.923 (with Yates' correction)	p<0.001
Any of the above	1 (1.15%)	53 (60.92%)	33 (37.93%)	87/450 (19.33%)	ꭓ^2^=235.676 (with Yates' correction)	p<0.001
None of the above	287 (79.06%)	76 (20.94%)	0 (0.00%)	363/450 (80.67%)		

## Discussion

This study examined the relationships between a range of sociodemographic risk factors, behavioral risk factors, medical conditions, events in life, and a previous history of depression or psychiatric issues with the depression levels experienced by older people living in the study area. The findings showed a significant relationship between several risk factors and the geriatric population's prevalence of depression in the aforementioned study area.

Sociodemographic factors and depression

In line with earlier research, older people had a higher prevalence of depression [16], and in the geriatric population, there was a strong relationship found between greater levels of depression and older age, lesser educational attainment, widowhood/divorce, lower income, and lower socioeconomic status [17,18]. These findings highlight the vulnerability of older adults, particularly those with limited resources and social support, to developing depression. Additionally, the lack of health insurance coverage was linked to increased severity of depression, which aligns with the findings of Langa et al. [19] and emphasizes the importance of access to healthcare services in mitigating mental health issues among the elderly.

Behavioral risk factors and depression

The study found a significant association between depression levels and a variety of behavioral risk factors. Lower levels of depression had a significant association with shorter sleep durations, both at night and during the day, which supports the findings of earlier research [20,21]. Tobacco and smoking addictions were also linked to increased depression severity, consistent with previous research [22,23]. In line with the conclusions of Daskalopoulou et al., a substantial risk factor for severe depression was shown to be a lack of physical activity [24], emphasizing the importance of promoting an active lifestyle among the elderly [25]. Alcohol addiction was another notable risk factor for depression, as reported by previous studies [20,21,26].

Medical conditions and depression

Higher levels of depression were significantly associated with a number of medical illnesses, including chronic constipation, asthma, heart disease, arthritis/musculoskeletal issues, visual impairment, and hypertension. These results align with earlier research [27-29] and draw attention to the reciprocal relationship that exists between elderly people's physical and mental health. It is even more crucial to address and manage chronic illnesses in this age group because the lack of comorbidities was linked to lower levels of depression.

Life events and depression

The research findings indicate a noteworthy association between a range of life events that occurred within the last year and increased levels of depression. An increased risk of depression has been associated with family conflicts, unemployment, illness (oneself or family members), death of family members or close relatives, big purchases or construction, and financial problems. These results are consistent with earlier research [30] and highlight how difficult life circumstances affect older people's mental health.

Limitations

Even though this study offers insightful information about the risk factors linked to depression in older people living in urban slums, there are several limitations that must be acknowledged.

Firstly, the study's cross-sectional design makes it impossible to demonstrate a causal association between the variables under investigation and depression severity. To investigate the temporal correlations and any reciprocal interactions between these variables and depressed symptoms over time, longitudinal research is necessary.

Secondly, self-reported data, on which the study relied on, may be prone to recall bias, especially for older people who may have cognitive impairment or who have had important previous life events. The accuracy of the data gathered may be improved by the use of clinical examinations and standardized assessment instruments.

Additionally, the study's exclusive focus on urban slum communities may have limited the findings' applicability to other senior groups living in other socioeconomic or geographic contexts. To investigate the possible differences in risk variables and their influence on depression in other population groups, more research is required.

Another drawback is the possible impact of unmeasured confounding factors, which could contribute to the onset and course of depression in older people. Examples of these factors include social support systems, coping strategies, and religious or cultural views. To obtain a fuller knowledge of the multidimensional complexity of depression within this subsection of the population, future studies could take these aspects into account.

Recommendations

Numerous suggestions for additional research, medical practice, and governmental initiatives can be made in light of the research's limitations and findings. First, it is important to carry out prospective and longitudinal research to determine the causal links among the identified risk variables and the initial or ongoing development of depression in elderly individuals. Second, comprehensive assessments that incorporate clinical evaluations, objective measures, and validated screening tools should be employed to enhance the accuracy of data collection and minimize potential biases. Third, research efforts should be expanded to include diverse elderly populations from various socioeconomic and geographic settings to improve the generalizability of findings and identify potential variations in risk factors and their impact on depression. Fourth, future research should examine the impact of undetected confounding factors on depression in older adults, including coping strategies, networks for social support, and religious or cultural views. Fifth, the modifiable factors influencing risk found in this study should be addressed by interventions and preventive measures, such as encouraging physical exercise, treating substance misuse, and offering assistance in managing long-term medical issues and major life events. Sixth, healthcare policies and programs should prioritize mental health services for the elderly, with a particular focus on vulnerable populations residing in urban slums or low-resource areas, ensuring access to screening, early intervention, and appropriate treatment options. Lastly, collaborations between researchers, healthcare professionals, policymakers, and community organizations should be fostered to develop comprehensive and culturally sensitive approaches to addressing depression in the elderly population.

## Conclusions

The current study contributes valuable insights into the factors associated with depression among elderly residents in urban slums, highlighting significant relationships between various sociodemographic, behavioral, medical, and historical factors and the prevalence and severity of depression in this population. The findings underscore the importance of addressing depression in older adults through a multifaceted approach, considering the complex interplay of risk factors. While limitations such as the cross-sectional design and reliance on self-reported data should be acknowledged, the results provide a foundation for developing targeted interventions, preventive strategies, and healthcare policies aimed at improving the mental well-being of vulnerable elderly populations. Future research should focus on longitudinal studies to establish causal relationships, explore potential confounding factors, and examine diverse elderly populations to enhance the generalizability and depth of understanding in this crucial area of public health.
